# DNA of *Brugia malayi* detected in several mosquito species collected from Balangan District, South Borneo Province, Indonesia

**DOI:** 10.14202/vetworld.2020.996-1000

**Published:** 2020-05-30

**Authors:** Supriyono Supriyono, Suriyani Tan

**Affiliations:** 1 Division of Parasitology and Medical Entomology, Faculty of Veterinary Medicine, IPB University, Bogor 16680, West Java, Indonesia; 2 Department of Parasitology, Faculty of Medicine, Trisakti University, Jakarta, Indonesia

**Keywords:** *Brugia malayi*, lymphatic filariasis, polymerase chain reaction

## Abstract

**Background and Aim:**

Lymphatic filariasis (LF) is a lesser-known parasitic disease, which contributes to significant decreases in overall health. This study investigated the presence of *Brugia malayi* in mosquitoes collected in the South Borneo Province, Indonesia.

**Materials and Methods:**

Mosquitoes were collected through bare leg collection methods after sunset in several areas of the Hukai and Gulinggang villages in the Balangan District. The collected mosquitoes were identified based on morphological features and dissected to find microfilaria and then pooled through species for polymerase chain reaction (PCR) microfilaria detection.

**Results:**

A total of 837 female mosquitoes consisting of at least 14 species were selected; they were dissected, and no microfilariae were found. Mosquitoes were divided into 69 pools for PCR analysis. PCR revealed that 8.7% (6/69) of the pools were positive for *B. malayi*, including *Mansonia annulifera* (4 pools), *Aedes albopictus* (1 pool), and *Culex tritaeniorhynchus* (1 pool).

**Conclusions:**

These results suggested that mosquito dissection was not an optimum method for finding microfilaria. *M. annulifera*, *C. tritaeniorhynchus*, and *A. albopictus* mosquitoes might play an important role in the transmission of LF in the Balangan District. Information from this study could be used for the prevention of transmission or vector control programs in Indonesia.

## Introduction

Lymphatic filariasis (LF) remains a public health problem and possesses a significant morbidity risk to a large proportion of low-income individuals and families in several countries [1,2]. At present, more than half of the world’s inhabitants reside in high-risk areas for this disease. Approximately 50% of infections are those who live in Southeast Asia, including Indonesia [3]. Once this parasite enters the host, the worms lodge in the lymphatic vessels of the host and causes swelling of the arms, legs, and genital organs, leading to debilitating impacts and suffering to the patient [4]. In 2000, the WHO strived to eliminate LF by announcing the Global Program to Eliminate LF and has targeted LF for elimination as a public health problem by the year 2020. Accordingly, the Indonesian Ministry of Health has applied two strategies. The first strategy is aimed at reducing and eliminating transmission by mass drug administration, while the second involves reducing and preventing morbidity in the affected persons [5]. These strategies gained little success; however, epidemiology data showed that in 2016, as many as 29 provinces were still considered endemic areas, with the highest prevalence rates in East Indonesia [6,7].

LF is a vector-borne disease caused by three species of tissue nematodes, *Wuchereria bancrofti*, *Brugia malayi*, and *Brugia timori*, all of which are endemic to several areas of Indonesia. This condition has complicated the elimination program in Indonesia, as it is the only country with all three species. The following are other challenges for LF elimination programs: (1) Indonesia is comprised of multiple Islands making the distribution of drugs uneven [8], and (2) the geography of Indonesia includes forest and paddy fields, which are suitable mosquito breeding grounds. Of the three main species, *B. malayi* alone accounted for 70% of the LF infection in Indonesia [9]. There are 23 species of mosquitoes, which have already been identified as vectors of LF from four genera (*Culex*, *Anopheles*, *Mansonia*, and *Aedes*). The zoophilic type of *B. malayi* is commonly found in Southeast Asia, including Indonesia; it involves animals as the reservoir host and is transmitted by *Mansonia* mosquitoes, while the anthropophilic type is transmitted by *Anopheles* [10]. Since the WHO supplemental strategy is to use the approach of vector control, we should fully understand the behavior of these mosquitoes. The Balangan District in South Kalimantan is one of the endemic areas of filariasis with forest, mountains, and swampy areas. The population was 112,430 inhabitants with the majority farmers of rubber plantations [11]. In 2014, the Balangan District Department of Health reported an outbreak of filariasis in several villages, such as Hukai and Gulinggang. The LF patients were generally diagnosed by Giemsa-stained blood smear examination. In 2015, 19% (4/21) of patients in the Balangan District were then reported positive with filariasis by polymerase chain reaction (PCR) [12].

The confirmation of vector capacity in microfilaria transmission is important for understanding the status of the disease. Until recently, the only way to measure the infectivity of mosquitoes was through the dissection method. However, dissection is not practical for detecting infection in mosquitoes, particularly when infectivity is low. PCR is a molecular approach that has the capacity for monitoring filariasis and may help to avoid premature cessation of the control programs. This study investigated the presence of *B. malayi* in mosquitoes collected in the South Borneo Province, Indonesia.

This study aimed to determine the potential vector of *B. malayi* through PCR to support the prevention and protection of the Balangan District, South Kalimantan, Indonesia, from a resurgence of filariasis.

## Materials and Methods

### Ethics approval

This research and all subjects involved in this research obtained an ethics letter from the Faculty of Trisakti University Medicine.

### Collection of mosquitoes

**Figure-1:**
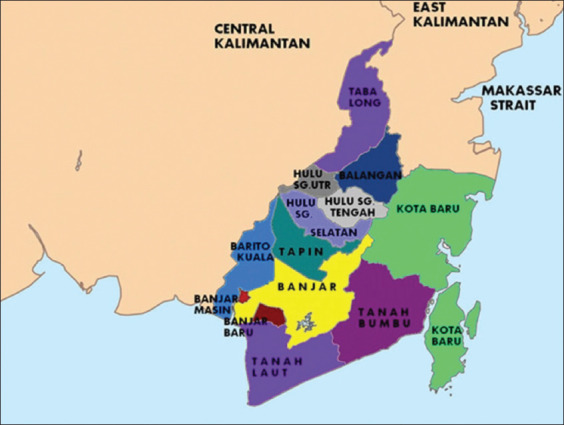
The map of Balangan District, South Kalimantan Province. (114°50’31 - 115°50’24 BT dan 2°1’31 - 2°35’58 LS). [Source of Latitude and Longitude: Government of South Kalimantan Province. Photo taken from www.infopendaki.com].

Adult female mosquitoes were collected by bare leg collection in several areas of the Hukai and Gulinggang villages in the Balangan District, Borneo Province (Figure-1) from January to March 2015. Leg collection involved the following: The catcher sat on a chair either indoors or outdoors with their bare legs exposed and caught landing mosquitoes through aspirator [13,14]. The collected mosquitoes were identified based on morphological features [15] and were then dissected to determine the infection rates of microfilaria. If no filaria was detected, the mosquitoes were pooled based on the species for detecting microfilaria through PCR analysis (Table-1).

**Table-1:** The collected mosquitoes, no. of pools and positive sample.

Mosquito species	Total mosquitoes	No. of pool	No. of positive pool
*Mansonia annulifera*	425	10	4
*Mansonia annulata*	120	29	0
*Mansonia bonneae*	53	7	0
*Mansonia uniformis*	17	2	0
*Mansonia dives*	8	1	0
*Aedes albopictus*	55	5	1
*Aedes.* spp.	5	ND	0
*Anopheles barbirostris*	12	1	0
*Anopheles letifer*	7	1	0
*Armigeres* spp.	36	3	0
*Culex tritaeniorhynchus*	50	5	1
*Culex quinquefasciatus*	34	3	0
*Culex gelidus*	14	2	0
*Aedes aegypti*	1	ND	ND
Total	837	69	6 (8.7%)

### DNA extraction

The DNA from the pooled mosquitoes was extracted by a GeneJET Genomic DNA Purification Kit (Thermo Fisher Scientific, USA), according to the manufacturer’s instructions. Detection of microfilaria was performed using a specific primer for *B. malayi* (Hha1 F 5′-GCG CAT AAA TTC ATC AGC-3′, Hha1 R 5′-GCG CAA AAC TTA ATT ACA AAA GC-3′, AIT biotech) [16]. PCR was conducted in 50 ml reaction mixtures with the master mix reagents for the PCR, which consisted of Taq DNA polymerase (0.05 U/mL), reaction buffer (4 mM MgCl_2_), dNTPs (0.4 mM each), and oligonucleotide primers (0.1 mM each). The thermal cycling conditions were 35 cycles at 95°C for 3 min followed by 95°C for 30 min, 57°C for 30 s, 72°C for 1 min, and a final extension step at 72°C for 1 min. PCR products were separated by electrophoresis on a 2% agarose gel, detected by SYBR staining, and visualized with a UVITEC Cambridge Gel Documentation system. DNA was determined to be microfilaria positive in our previous study served as positive control.

## Results

**Figure-2:**
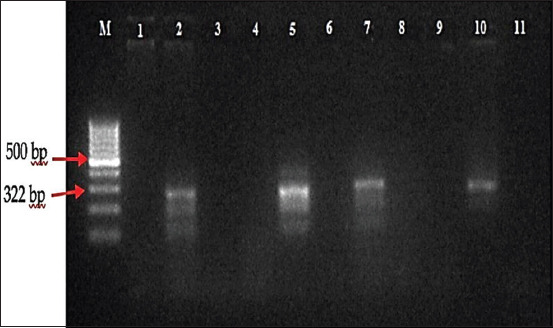
Polymerase chain reaction profile of *Brugia malayi* from mosquitoes collected in Gulinggang village. M: DNA Marker/*ladder* 100 bp, column 1-2: Pools of *Mansonia annulifera*, column 3: Pool of *Ma. bonneae*, column 4: Pool of *Mansonia annulata*, column 5: Positive control, column 6-7: Pools of *Mansonia annulifera*, column 8: Pool of Nyamuk *Mansonia bonneae*, column 9: Pool of *Mansonia annulata*, column 10: Positive control and column 11: Negative control.

**Figure-3:**
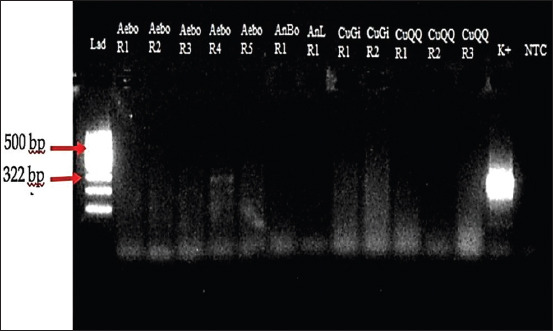
Polymerase chain reaction profile of *Brugia malayi* from mosquitoes collected in Gulinggang village. Column Lad: DNA Marker, column Aebo R1 – R5: Pool of *Aedes albopictus*, column AnBo R1: Pool of *Anopheles barbirostris*, column AnL R1: Pool of *Anopheles letifer*, column CuGi R1–R2: Pool of *Culex gelidus*, column CuQQ R1–R3: Pool of *Culex quinquefasciatus*, column K+: Positive control, and column NTC: Negative control.

**Figure-4:**
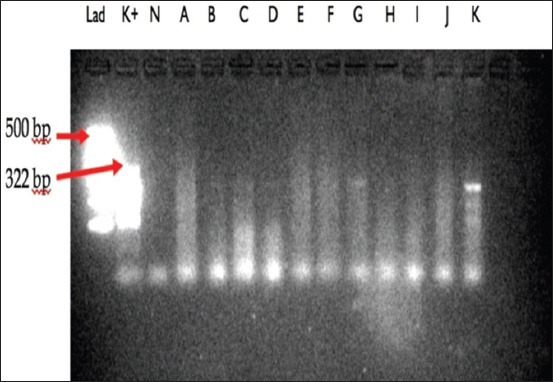
Polymerase chain reaction profile of *Brugia malayi* from mosquitoes collected in Gulinggang and Hukai villages. Column Lad: DNA Marker, column K+: Positive control, column N: Negative control, column A: Pool of *Aedes albopictus*, column B–D: Pool of *Armigeres* sp. and column E–K: Pool of *Mansonia annulifera*.

**Figure-5:**
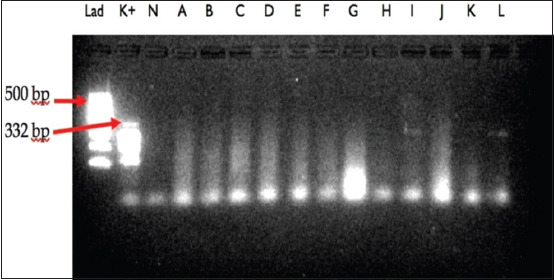
Polymerase chain reaction profile of *B. malayi* from mosquitoes collected in Gulinggang and Hukai villages. column Lad: DNA Marker, column K+: positive control, column N: negative control, column A–G: pool of *Ma. annulata*, column H–L: pool of *Cx. tritaeniorhynchus* and column E–K: pool of *Mansonia annulifera*.

In this study, 837 selected female mosquitoes that consisted of at least 14 species were divided into 69 pools ranging between 7 and 43 mosquitoes (Table-1). All selected female mosquitoes were dissected, and no microfilaria was found. The mosquitoes were then subjected to a determination of microfilaria by PCR, and amplification of the *B. malayi* microfilariae DNA using a species-specific primer was demonstrated by the presence of a 322-bp PCR product (Figures-2-5). PCR investigation revealed that 8.7% (6/69) of the pools were positive for *B. malayi*, such as *Mansonia annulifera* (4 pools), *Aedes albopictus* (1 pool), and *Culex tritaeniorhynchus* (1 pool).

## Discussion

PCR diagnosis was used for screening of *B. malayi* in the Balangan District, South Borneo Province. The selected female mosquitoes that were reported negative of microfilaria infection by microscopy methods were used for molecular analysis. The PCR method was found to be significantly more sensitive compared to microscopy in detecting the filarial parasite in the collected mosquito samples. Our previous study indicated that no mosquitoes were found to be infected with microfilaria when dissected [14]. In this study, four out of ten pools of *M. annulifera* were positive for *B. malayi*. Several studies have revealed that the genus *Mansonia* was reportedly the main vector of the zoophilic type of *B. malayi* in several areas in Indonesia, such as the South Sumatra and Jambi Provinces, Sumatra Island [9,17].

Interestingly, in this study, we found a positive sample for both *A. albopictus* and *C. tritaeniorhynchus* collected from the Gulinggang village. Since *A. albopictus* and *C. tritaeniorhynchus* were not previously considered primary vectors of *B. malayi*, this result suggested that these mosquitoes might play an important role in transmitting *B. malayi* in the Balangan District. A study in India showed that the genus *Culex* was considered to be the main vector of the filarial parasite *W. bancrofti* that causes human LF [18], while the genus *Aedes* particularly *Aedes aegypti* was not found to be an efficient vector of *B. malayi* [19]. Since this study used the bare leg method for mosquito collection, we hypothesized that the presence of the specific 322 bp bands found in the pools of *A. albopictus* and *C. tritaeniorhynchus* came from mosquitoes that had just fed on the blood of infected subjects. Gulinggang and Hukai villages are close to a big open swamp and irrigated fields and surrounded by rubber plantations. There were many aquatic plants in the swamp; therefore, it was full of potential breeding places for mosquitoes, particularly the genus *Mansonia*. A previous study revealed that swamps with many aquatic plants are the ideal breeding places for *Mansonia* spp. [20]. In addition, most of the people in these villages were farmers of rubber plantations. Thus, their activities in the rubber plantation after sunset exposed them more frequently to infected mosquitoes and increased the possibility of contracting *B. malayi*. Our previous reports also indicated that several cats in Gulinggang village were found to be positive for *B. malayi* [13] and four out of 21 persons in Balangan District who had received diethylcarbamazine for 10 days exhibited positive DNA for *B. malayi* by PCR [12]. *B. malayi* has been known to have multiple definitive hosts, such as humans, monkeys, domestic cats, or forest carnivores. A limitation of this study was that we could not differentiate between the infective and infected mosquitoes, as the head, thorax, and abdomen were not tested separately. However, we successfully detected the presence of DNA for *Brugia* spp. in *M. annulifera*, *C. tritaeniorhynchus*, and *A. albopictus* by PCR. The existence of multiple mosquito vectors combined with abundant breeding places has allowed the reservoir in these villages to be maintained; therefore, the potential transmission of *B. malayi* might become a future threat to human health.

## Conclusion

While the genus *Mansonia* remains the main vector in the Balangan District, South Borneo Province, this study showed that additional research is required on another genus of mosquitoes as they might serve as alternative vectors for *B. malayi*. These results should impact future vector control programs in Indonesia, as the control program should not focus only on the main vector, and it should be applied based on the results of future bionomic studies on mosquito vectors.

## Authors’ Contributions

SS and ST designed the experimental protocol. ST collected and analyzed the samples. SS and ST drafted and corrected the manuscript. Both authors read and approved the final manuscript.

## References

[1] Otsuji Y (2011). History, epidemiology and control of filariasis. Trop. Med. Health.

[2] Boko-Collins P.M, Ogouyemi-Hounto A, Adjinacou-Badou E.G, Gbaguidi-Saizonou L, Dossa N.I, Dare A, Ibikounle M, Zoerhoff K.L, Cohn D.A, Batcho W (2019). Assessment of treatment impact on lymphatic filariasis in 13 districts of Benin:Progress toward elimination in nine districts despite persistence of transmission in some areas. Parasit. Vectors.

[3] Specht S, Suma T.K, Pedrique B, Hoerauf A.A (2019). Elimination of lymphatic filariasis in South East Asia. BMJ.

[4] Allen T, Taleo F, Graves P.M, Wood P, Taleo G, Baker M, Bradley M, Ichimori K (2017). Impact of the lymphatic filariasis control program towards elimination of filariasis in Vanuatu, 1997-2006. Trop. Med. Health.

[5] Titaley C.R, Damayanti R, Soeharno N, Mu'asyaroh A, Bradley M, Lynam T, Krentel A (2018). Assessing knowledge about lymphatic filariasis and the implementation of mass drug administration amongst drug deliverers in three districts/cities of Indonesia. Parasit. Vectors.

[6] Wibawa T, Satoto T.B.T (2016). Magnitude of neglected tropical diseases in Indonesia at postmillennium development goals era. J. Trop Med.

[7] Lee J, Ryu J.S (2019). Current status of parasite infections in Indonesia:A literature review. Korean J. Parasitol.

[8] Tan M, Kusriastuti R, Savioli L, Hotez P.J (2014). Indonesia:An emerging market economy beset by neglected tropical diseases (NTDs). PLoS Negl. Trop. Dis.

[9] Mulyaningsih B, Umniyati S.R, Hadisusanto S, Edyansyah E (2019). *Mansonia uniformis*:A locally important vector of *Brugia malayi* nocturnally sub-periodic type in South Sumatera of Indonesia. Southeast Asian J. Trop. Med. Public Health.

[10] McNulty S.N, Mitreva M, Weil G.J, Fischer P.U (2013). Inter and intra-specific diversity of parasites that cause lymphatic filariasis. Infect. Genet. Evol.

[11] (2020). Government of Balangan District.

[12] Tan S, Supriyono S, Ullyartha H (2016). Comparison of microscopic to PCR for detecting microfilaria in 21 lymphatic filariasis patients treated with diethylcarbamazine. Indones. J. Biomed. Sci.

[13] Supriyono, Tan S, Hadi U.K (2017). Behavior of *Mansonia* and potency of reservoir on transmitting of filariasis in Gulinggang village Balangan District South Kalimantan Province. J. Aspirator.

[14] Supriyono, Tan S, Hadi U.K (2019). Mosquito diversity and habitat characteristic in Juai Subdistrict, Balangan District, South Kalimantan Province. J. Aspirator.

[15] Rampa R (1982). A guide to the genera of mosquitoes (*Diptera*
*Culicidae*) of Thailand with illustrated keys, biological notes and preservation and mounting techniques. Mosq. Syst.

[16] Saeed M, Siddiqui S, Bajpai P, Srivastava A.K, Mustafa H (2014). Amplification of *Brugia malayi* DNA using Hha1 primer as a tool. Open Conf. Proc. J.

[17] Santoso S, Yahya Y, Suryaningtyas N.H, Rahayu K.S (2015). Dissection and PCR-based detection of *Brugia malayi* on *Mansonia* spp in Tanjung Jabung Timur District. J. Aspirator.

[18] Shriram A.N, Krishnamoorthy K, Vijayachari P (2015). Diurnally subperiodic filariasis among the Nicobarese of Nicobar district epidemiology, vector dynamics and prospects of elimination. Indian J. Med. Res.

[19] Ariani C.V, Juneja P, Smith S, Tinsley M.C, Jiggins F.M (2015). Vector competence of mosquitoes for filarial nematodes is affected by age and nutrient limitation. Exp. Gerontol.

[20] Sapada I.E, Anwar C, Priadi D.P (2015). Environmental and socio-economic factors associated with cases of clinical filariasis in Banyuasin district of South Sumatera, Indonesia. Int. J. Collab. Res. Intern. Med. Public Health.

